# Attitudes towards the use and acceptance of eHealth technologies: a case study of older adults living with chronic pain and implications for rural healthcare

**DOI:** 10.1186/s12913-015-0825-0

**Published:** 2015-04-16

**Authors:** Margaret Currie, Lorna J Philip, Anne Roberts

**Affiliations:** Social, Economic & Geographical Sciences, The James Hutton Institute, Craigiebuckler, Aberdeen, AB15 8QH UK; Geography & Environment, School of Geosciences, University of Aberdeen, Elphinstone Road, Aberdeen, AB24 3UF UK; The Centre for Rural Health, University of Aberdeen, The Centre for Health Sciences, Aberdeen, IV2 3JH UK

**Keywords:** Rural health, Chronic pain, eHealth, Service delivery, Older adults, Technology

## Abstract

**Background:**

Providing health services to an ageing population is challenging, and in rural areas even more so. It is expensive to provide high quality services to small populations who are widely dispersed; staff and patients are often required to travel considerable distances to access services, and the economic downturn has created a climate where delivery costs are under constant review. There is potential for technology to overcome some of these problems by decreasing or ceasing the need for patients and health professionals to travel to attend/deliver in-person appointments. A variety of eHealth initiatives (for example Pathways through Pain an online course aimed to aid self-help amongst those living with persistent pain) have been launched across the UK, but roll out remains at an early stage.

**Methods:**

This mixed-methods study of older adults with chronic pain examines attitudes towards, current use of and acceptance of the use of technology in healthcare. A survey (n = 168, 40% response rate) captured broad experiences of the use of technology in health and social care. Semi-structured interviews (four *with technology* and seven *without technology* participants) elicited attitudes towards technology in healthcare and explored attributes of personal and social interaction during home visits.

**Results:**

People suffering from chronic pain access healthcare in a variety of ways. eHealth technology use was most common amongst older adults who lived alone. There was broad acceptance of eHealth being used in future care of people with chronic pain, but older adults wanted eHealth to be delivered alongside existing in-person visits from health and social care professionals.

**Conclusions:**

eHealth has the potential to overcome some traditional challenges of providing rural healthcare, however roll out needs to be gradual and begin by supplementing, not substituting, existing care and should be mindful of individual’s circumstances, capability and preferences. Acceptance of technology may relate to existing levels of personal and social contact, and may be greater where technological help is not *perceived* to be replacing in-person care.

**Electronic supplementary material:**

The online version of this article (doi:10.1186/s12913-015-0825-0) contains supplementary material, which is available to authorized users.

## Background

Providing health services in rural areas is challenging: it is expensive to provide services to dispersed populations; there is an increasing proportion of older people in the population as a whole, a sizeable minority of whom have complex health needs; staff and patients routinely travel considerable distances to either access or deliver services; and fiscal constraints in the public sector associated with the economic downturn have created further financial pressures that require health and social care service providers to identify new, more efficient delivery models. There is considerable potential for new and existing technologies to be used to overcome some of these challenges. Despite the upbeat rhetoric used by advocates of eHealth^a^, deployment of technology remains at a very early stage. Improving our understanding of patients’ and health professionals’ attitudes towards the use and acceptance of technology, applied in personal and ‘medicalised’ or professional lives can contribute to further developments in the use of eHealth as part of a care package. In this paper we report a 2-stage study in which attitudes towards, existing use of and acceptance of technology (such as eHealth and the Internet, for example) in rural health care is explored with a particular focus on the experience of rural adults living with chronic pain.

The study focused on older adults (aged 60+) with chronic pain. The incidence of chronic pain increases with age [1] and it is important to understand the experiences and attitudes of older adults living with chronic pain to better inform future health service planning. There is a requirement to be cognisant of the impacts demographic ageing will have in healthcare demands whilst simultaneously recognising that attitudes to technology may change in future generations of older adults.

In this paper we use the term ‘technology’. By technology we mean everyday digital technologies including devices that people use to keep in contact with others, such as a mobile (or cell) phone or a smart phone, a computer or an Internet-enabled television and the various applications that can be used on these devices such as: email, voice-over-Internet services such as SKYPE, and social networking sites. Technology in the form of digital devices and/ or applications can also be used as part of a health and social care package. We refer to such technologies as eHealth. eHealth itself is a broad concept that covers a plethora of themes with differing definitions [2]. However, two key themes are health and technology thus eHealth encompasses telehealth and telecare technologies but can also include online courses (for patients and professionals) and patient-health professional consultations. Telecare involves monitoring aspects of an individual’s activity, or related activities, in the home e.g. fall alarms and motion sensors. Telehealth technologies require active involvement from the patient to take readings e.g. blood pressure, that are regularly submitted for review by health professionals (for more examples see [1]). Peoples’ willingness to use everyday technology may be indicative of their likely receptiveness to eHealth now and/ or in the future.

eHealth has the potential to overcome some service delivery challenges in rural areas, where the proportions of older people are increasing more rapidly than in urban areas [1]. However any implementation of eHealth needs to be sensitive to the needs of patients, their care package (i.e. all the elements of care an individual receives) and the attributes of the geographical area in which they live. Others have found that the development of technological support for older people needs to include the user’s perspective [3]. This study thus offers a timely exploration of attitudes towards, use of and acceptance of technology (such as eHealth, the Internet, the use of smart phones with ‘medical’ applications) in rural settings, with a particular focus on the experience of adults living with chronic pain. The findings contribute to ongoing debates about service delivery reform.

Chronic pain, is defined by the Pain Society as “Continuous, long-term pain of more than 12 weeks or after the time that healing would have been thought to have occurred in pain after trauma or surgery” [4] and is the most common symptom of disease. The chronic pain group is large: it has been estimated that chronic pain affects 14% of the UK population [5]. It is associated with a diverse range of health conditions (for example, arthritis, multiple sclerosis, cancer and diabetes) and thus those living with chronic pain, and the ways in which it affects their lives, will vary. Chronic pain is reported to affect physical and psychological health (see, for example, [6,7]) and chronic pain patients access health services up to five times more often than any other group [8,9]. It is associated with social isolation because sufferers may find it both difficult to get out of their homes [10] and challenging to spend periods of time interacting with other people. The incidence is highest in rural areas [11-14].

Adults with chronic pain are routinely encouraged to adopt self-management or an ‘enabled’ approach to deal with their condition. The potential of eHealth to facilitate pain management activities, and to promote self-management of the condition, has been identified in the UK (e.g. the NHS *Pathway through Pain* programme, see [15]). The Pathway through Pain programme is used in this paper as an example of an eHealth application. These and other related types of eHealth applications could be particularly useful to the rural population for whom accessing pain services and support groups in person is often difficult. For example, pain clinics are often located in District General hospitals or nearer larger urban centres which are hard to access by rural patients.

The prevalence of chronic pain is known to increase with age and then plateau in the 65+ age group [16]. Increasing numbers of older people in national populations in Europe and North America are already placing demands upon health and social care services, especially in rural areas, which have notably older demographic profiles than urban areas and are forecast to age faster in the foreseeable future [17]. Novel ways of providing care to an already large patient group are attractive from a service provider perspective. From a patient perspective, decreasing, or ceasing the need to travel to attend health care appointments is also likely to be attractive, especially to those who live at a distance from large urban centres with specialist services. However, for increased usage of eHealth to be successful we need to understand patients’ and health professionals’ attitudes towards accessing health services in this way and use this information in the design, development and evaluation of eHealth initiatives. If patients and health professionals are not receptive to an eHealth initiative there is a risk of less or un-successful deployment of that initiative.

For eHealth to be a viable service delivery option in rural healthcare, it is important to understand both its potential and barriers to its use. If patients and staff are not technologically literate, eHealth implementation will not be successful. In 2011, almost a quarter of adults in EU member states had never used the Internet [18]. The likelihood of an individual to have never used the Internet increases sharply with age but the proportion of adults aged 65–74 who had never used the Internet across Europe dropped considerably between 2005–2011 [18]. UK data [19] show that there are no urban–rural differences in the trend of increasing proportions of older Internet users. ‘Younger’ older people in the UK are more than twice as likely to be Internet users than ‘older’ older people [19]. As the ‘young old’ age, digital literacy in the ‘older old’ cohorts of the future will increase. Thus while statistics highlight the digital exclusion of many older adults, they suggest that age-based disparities in Internet use are reducing considerably. We may assume that increasing numbers of older adults will be users of the types of technologies required to access eHealth services in the near future and postulate that older adults will be accepting of such technologies as part of their care package.

Some eHealth initiatives have already been launched in the UK and are designed to bring both benefits to patients and to deliver significant savings to the health budget (for example, for England see [20]) and greater use of eHealth solutions in the delivery of care for older people has been explicitly recommended by the Scottish Public Health Network [21]). However, deployment of eHealth remains at an early stage. Roll out in many rural communities must overcome capacity issues relating to the digital infrastructure. The needs and preferences of the people eHealth seeks to benefit must also be incorporated into eHealth strategies.

Within the context of chronic pain - which is very common - there is potential for eHealth to become a routine element of managing the condition. Thus chronic pain patients were selected as the group to focus on for this research. To consider the potential use of technology in delivering healthcare to people in rural areas we need to consider what people already use technology for, including health related devices and applications, and the reasons they do this; explore what technology they might be willing to use or be interested in either for self-management of their health condition or as part of their formal care package; and reflect on how expectations and use of technology might change in the medium term. The research thus presents an opportunity to explore some of the opportunities and challenges of rolling out eHealth into the management of chronic pain, particularly in rural areas.

## Methods

A 2-stage research design was adopted. Stage 1 used a postal questionnaire survey to elicit attitudes, opinions and experiences from the membership of Pain Association Scotland, a voluntary organisation that provides self-management training for individuals with chronic pain and which addresses the non-medical aspects of living with chronic pain in an attempt to improve the quality of life of pain sufferers. Stage 2 comprised interviews with older adults with chronic pain who received regular home visits from health and/or social care professionals, interviews with professionals who delivered in-person care in the homes of chronic pain patients, and interviews with the small numbers of older adults who had completed the online Pathway through Pain programme in Scotland. This approach allowed the experiences of older adults with differing levels of pain severity, impairment and levels of engagement with health care services to be collected^b^.

### Questionnaire

The questionnaire was developed by the authors with input from other members of the *Technology for Older Adults: Maximising Personal and Social Interaction* (TOPS) research team. The questions were specifically designed to answer TOPS research questions and included open and closed response and likert scale questions. Two previously validated question sets -EQ5D and SQ12- were also included. Surveying the membership of Pain Association Scotland - the only Scotland-wide voluntary organisation that is exclusively concerned with chronic pain - represented a purposive approach to eliciting attitudes and opinions from adults living with chronic pain. The questionnaire was administered to all (n = 425) members of Pain Association Scotland. It asked for information about respondents (age, gender, marital status, educational attainment leave, living arrangements, proximity of relatives and friends); their health, life satisfaction and the nature of their interactions with health and social care providers and informal care and support arrangements with family and friends. Finally a number of questions about availability, attitudes and acceptance towards technology were asked, including use of widely available technologies (e.g. email, mobile phone), the use of assisted living devices in the home (e.g. fall alarms) and attitudes towards and preferences regarding use of telemedicine and communication technologies in the provision of healthcare now and on the future. Respondents were requested to report their full postcode in order that their responses could be geocoded according to the Scottish Government’s Urban–rural classification.

In November 2012, the Pain Association Scotland posted the questionnaire on our behalf, ensuring their membership information remained confidential. Following a reminder sent in January 2013, 168 useable responses were returned, a response rate of 40%.

### Interviews

The second stage of the study, a series of qualitative semi-structured interviews, focused explicitly on older adults (aged 60–75) who lived in rural areas and who had long-term chronic pain. Two separate groups were interviewed, a *without technology* group and a *with technology* group. Interviews with the *without technology* group were preceded by the observation of a home visit and followed by interviews with the health or social care professional attending the home visit. During all the interviews older adults and professionals were asked to reflect on their experiences of and attitudes towards their existing and potential future use of technology in both their private and their ‘medicalised’ or professional lives.

The *without technology* group involved (i) older adults who received regular home visits from health and/or social care professionals and who did not use any eHealth technology in the management of their chronic pain and (ii) the professionals who delivered care to those older adults in their homes. Interviews were conducted in a very remote island community in Scotland, selected for the study following detailed discussions with the lead clinician for chronic pain in the NHS Highland area during which it was ascertained that there was a cluster of patients living on the island who met the TOPS project selection criteria. Older adults who met the inclusion criteria of the study (living in a rural area, aged 60 to 74, living independently in their own home, and receiving regular home visits from health and/ or social care professionals) were identified and locally following conversations with professional gatekeepers on the island who comprised GP Practice Managers, community nursing team members and the island’s Social Care Team. The numbers interviewed were determined by those who met the inclusion criteria and who were considered suitable participants (i.e. able to give informed voluntary consent to participate in the study). Seven individuals were identified and all agreed to participate in the study. The seven participants were all severely affected by their chronic pain and had limited personal mobility. Five professionals also participated in the study.

The *with technology* group who were interviewed for the study comprised the small number of older adults living in a rural area of Scotland who had personal experience of using eHealth in the management of their chronic pain. All members of this group had recently completed, the *Pathway through Pain* programme. This is a UK-wide, on-line pain self-management programme [15] currently offered by 8 NHS Boards^c^, including 2 in Scotland. Patients are referred by their GP and go through a 24 step programme in which they are introduced to different techniques that can be used in the self-management of chronic pain. A small number of patients in one Scottish NHS board area - NHS Tayside - have completed the online *Pathway through Pain* programme. Six met the criteria for participation in the study (living in a rural area, aged 60 to 74, living independently in their own home, and not receiving regular home visits from health and/ or social care professionals) and four agreed to be interviewed about their experiences of participating in the programme. This group of interviewees were not as severely afflicted as those in the *without technology* group. Their personal mobility was not overly constrained by their condition and they did not require regular home visits to manage their chronic pain.

### Analysis

Stage 1 report descriptive statistics and the findings from a series of tests of association appropriate for the analysis of non-parametric, categorical data (Chi-square). SPSS version 21 was used to perform the analysis. Stage 2 adopted an iterative framework approach [22] for the analysis of interview transcripts, an approach that involves familiarisation with the data, identification of a thematic framework, indexing, charting, and finally mapping and interpreting the findings. Transcripts were coded independently by three members of the TOPS research team and the coding framework was then developed collaboratively. All interview transcripts and field notes were managed and analysed in NVivo 9. The study is thus mixed-methods in style, with findings from quantitative Stage 1 and qualitative Stage 2 being compared to identify complementary findings which provide a greater understanding of the issues being examined than would be possible if only one method had been used [23].

## Results

We now present findings from Stage 1 (questionnaire) and Stage 2 (interviews). Stage 1 findings set out broad attitudes towards, and acceptance of technology by older adults living with chronic pain in Scotland, focusing on the types of help received by respondents, their current use of technology and their attitudes towards future use of technology in the home. Stage 2 focuses more specifically upon the rural experience and identifies both how older adults are using technology to manage chronic pain and some of the problems faced when using technology in rural areas.

### Results from stage 1 (questionnaire)

Completed questionnaires were received from 168 individuals whose key attributes are described below in Table 1. Unfortunately the attributes of the Pain Association membership (age, gender etc.) are not known; the organisation does not keep such information thus it is impossible to ascertain the representativeness of the sample. Approximately one third of respondents were men and two thirds were women. Although respondents ranged in age from 25 to 86 only 20% were aged under 50. Just over a half (54%) were aged 60+. The age distribution is thus consistent with the relationship noted above between incidence of chronic pain and age. 16.2% of respondents lived in a rural area, a figure slightly lower than the national average (18% of the Scottish population live in rural areas which cover 94% of the country’s land mass [24]).

**Table 1:** **Key descriptive information about respondents to the survey**

**Independent variables**	**Description of attributes of survey respondent**
Sex	36.5% (n = 61) were male and 63.5% (n = 106) were female
Age	Range = 25 to 86 years; mean = 60 years; median = 61 years
Number in household	33.3% (n = 56) were single person households; 46.4% (n = 78) were two person households; 21.3 (n = 31) lived in households with at two or more other people.
Urban or Rural	16.2% (n = 27) of the sample were rural respondents
Marital/Civil Status	Married (52.4%, n = 88); Single - never married (16.7%, n = 28); Separated/Divorced/Widowed (25%, n = 42)
Qualifications	O-level or equivalent (20.6%, n = 29); Degree (14.9%, n = 21)
Employment	Full time employment (19.5%, n = 16); Retired (55.4%, n = 93)

Questionnaire data were analysed to explore attitudes towards and acceptance of technology. Non-parametric tests of association were computed for a group of independent variables to ascertain firstly the types of formal and informal help respondents were currently receiving and, secondly, their current use of technology and attitudes towards using technologies to managing their chronic pain in the future. Results of statistical tests that were statistically significant at 95% are considered below. Due to the small sample size, we also highlight findings in Additional file 1: Table S1 and Additional file 2: Table S2 that were statistically significant at 90% (all identified with ‘*’ in the text) because they suggest relationships which could be important and would be worth further exploration.

#### Types of help received by respondents

Respondents were classified as receiving formal help if they stated they had received an in-person visit, in their home, from at least one of the following: a home help; community or district nurse; GP; other health professional. Additional file 1: Table S1 shows that people who were receiving formal help were more likely to be older, living alone (i.e. had always lived alone)*, dissatisfied with life, and not living with a partner or spouse (i.e. widowed or separated/ divorced). People in receipt of informal help, from family, friends, neighbours etc., were more likely to live in a household with other people*, be permanently sick*, and not have a partner or spouse (i.e. be widowed or separated/ divorced). Respondents who stated that they currently used technology in their home to manage or monitor their condition (e.g. fall alarms, types of monitors that send clinical information (e.g. blood pressure) to a health professional) were classified as being in receipt of technological help. These respondents were most likely to be older*, living alone*, in receipt of formal help and retired.

These findings suggest that there are relationships between being older, living alone (had always lived alone) and being without a partner or spouse (living alone due to widowhood or being separated/ divorced) and the types of care people are receiving. Respondents with these attributes receive the most formal help, which suggests that adults with chronic pain who live with other people rely on the other member of their household to help them and to meet their day-to-day care needs. Respondents defined as receiving technological help were also likely to be receiving formal, in-person, help. The findings also highlight the important role of informal help from family and friends, particularly informal care provided by other members of the household.

#### Current use of, and attitudes towards future use of, technology in the home

The questionnaire included questions designed to elicit information about respondents’ current use of technology (devices such as mobiles phones and computers and applications such as email, and social networking and using the Internet as a source of information) and attitudes towards the future use of technology in the home. Significant test results relating to these questions are reported in Additional file 2: Table S2.

The ways in which everyday technologies were used was similar for all respondents. However, respondents most likely to state that they used the Internet to monitor or manage aspects of their health were younger and not retired. Respondents who stated that they were happy to consider using technology in the future were more likely to be male and those who had no relatives living locally. Respondents who were living alone were less accepting than those who lived with other people to have some or all of the formal help they received replaced by technology. There were no differences found between the independent variables for acceptance of informal care being provided using technology. We return to these important findings in the discussion section.

### Results from stage 2 (interviews)

All the older adults that were interviewed – in both the *with* and *without technology* groups - had personal experience of using information and communications technologies in their everyday lives, including mobile telephones, personal computers, laptops and tablets (note that experience of using technology was not a criterion for inclusion in the study). Two participants used SKYPE to keep in touch with friends and family, others were regular Internet users and one participant told us about using the Internet to find out more about their medical condition. The interviewees are part of the growing proportion of the UK’s older population who are regular users of ICT.

Difficulties in using technology were reported by interviewees in the *without technology* group who noted that digital infrastructure deficiencies, ergonomic challenges and specific aspects of their medical condition affected their use of technological devices. Health professionals, whilst supportive of the incorporation of eHealth into care packages for older adults with chronic pain and other long term limiting illnesses recognised that it was important to consider an individual’s personal circumstances when considering further use of eHealth technologies. The older adults in the *with technology* group, who had completed the *Pathway through Pain* programme and had mixed views about it, particularly in terms of the balance between an online self-management programme and other methods of supporting the self-management of chronic pain. These issues are all relevant to the future design and development of eHealth technologies for older people.

#### Using technology to manage chronic pain

The *with technology* group comprised three males and one female who suffered from a variety of chronic conditions that resulted in episodes of acute or persistent chronic pain. The impact chronic pain had on their daily lives varied. For example, *with technology* Interviewee 4 was in constant pain and found it difficult to walk any distance, telling us “*I’m finding it very hard to walk any distance at all and I’m really struggling at work to keep going”.* Another interviewee was able to be active most of the time, dog walking and playing golf regularly. The *with technology* interviewees had all made use of a variety of NHS (e.g. GP, physiotherapist) and private (e.g. physiotherapist, osteopath, chiropractor) health services and, with the exception of using TENS machines which were self-identified as a type of eHealth, did not report the use of eHealth devices. Participation in *Pathway through Pain* was thus their only experience of using an eHealth technology.

Two interviewees had attended an in-person *Pathway through Pain* programme, delivered via local group meetings, and then followed this with the online course. The other two only had experience of the online course. There was no consensus regarding a preference for online or in-person delivery of the Pathway through Pain programme. The fact that the online course could be completed at one’s own pace, on one’s own was liked and one interviewee told us that they hadn’t liked attending a group meeting, much preferring the online course:

“*I wasn’t really impressed. Nothing to do with the people who were running it, just didn’t like being in amongst people … Some of them were needing to be there because they were obviously in wheelchairs and things like that, that sort of level of pain, or illness, and I didn’t feel that I quite belonged there somehow*” (*with technology* Interviewee 2).

Attending group meetings and participating face-to-face in fixed-time meetings required a more ‘structured’ commitment to the programme: whilst this will suit some people the flexibility of online participation is preferred by others. Social aspects of the group meetings were mentioned positively, perhaps most important for older adults who live on their own or whose chronic pain restricts their mobility, preventing them from getting out and about and interacting with other people as much as they would like to. We were told:

“*I much preferred the face to face group. … I don’t stay indoors all the time - but there were people there who didn’t get out a lot and they found it very good to be meeting others. That was the big thing, meeting people who were in the same boat as you, if you like, felt that pain every day and how they cope with it*” (*with technology* Interviewee 3).

*With technology* interviewees liked the fact that the individual steps of the *Pathway through Pain* programme were delivered by different people, from different professions. It would be difficult to achieve such multi-specialist input to a programme delivered in person anywhere but large population centres where specialists tend to be based. All the interviewees reported that the *Pathway through Pain* programme had introduced them to new relaxation techniques and to new exercises to do at home (the exercises were still being used). The resource materials (which include audio and video files) available to them during the course were commended. One interviewee told us that he had a copy of one of the relaxation files on his computer and used it regularly. Another interviewee told us that having completed the programme, they could not return to elements of it, such as resource materials:

“*… I couldn’t go back and do the online thing again, no, it seemed to vanish after a certain time. It was there on your machine, on my computer for a few months I think and then it just vanished, it went. But it gave you a warning saying it was leaving”* (*with technology* Interviewee 3).

Pathway through Pain organisers confirmed that patients *could* return to resource materials. Therefore there are misunderstandings between users and service providers which should be addressed. An ability to return to elements of the programme, such as the exercise instructions, would be particularly useful for patients who live in areas (including most remote rural areas) where a physiotherapy service is not available locally.

None of the *with technology* group had experienced any difficulties in using the *Pathway through Pain* eHealth application. Their comments suggest that, providing the user has very basic ITC skills, they should be able to participate in the programme.

“*Beginners could operate that, no bother … Anyone could do it!*” (*with technology* Interviewee 2).

The importance of personal attributes to determining how useful the programme would be were also mentioned. “*It’s up to the person who is watching and listening to get something out of it*” (*with technology* Interviewee 4). Health professionals also identified this as a requirement for eHealth to be successful.

#### Problems using technology in rural areas by chronic pain patients

Technology challenges associated with the capabilities of an individual to use technology and broader infrastructure challenges are now considered. Ergonomic challenges, difficulties caused by eyesight and hearing impairments and the lack of concentration, becoming tired easily and symptoms of pain, were all mentioned. Hearing impairments make it difficult to participate in an online patient support group:

“*I find with my hearing it’s very difficult, I couldn’t do a group, it would have to be one or two at the most …*” (*without technology* interviewee 4).

Another interviewee found using her tablet much easier than a laptop. She couldn’t use the latter’s tracker pad and having to use a mouse limited where she could sit and use the laptop. She said of her iPad “ *… I just sit on my bed with my knees up, not holding it, like this … cos I couldn’t I just rest it. … It’s much easier on the iPad*” (*without technology* Interviewee 6). For *without technology* interviewee 1, the fact that interacting with other people, face-to-face or otherwise (by phone or Skype for example), was very tiring. She found this frustrating: “*It exhausts me. Not as bad just now but it’s still … you are limited to how long you can talk. How long you can listen, that’s part of it*”.

A considerable challenge faced by most remote rural communities across the UK is slow and unreliable broadband services. Mobile Internet access (3G or 4G signal) is not available in many rural areas and download speeds on fixed Internet connections tend to be much slower in rural compared to urban areas (see data published by Ofcom, the UK telecommunications regulator). This means that, in rural areas, it can be very slow to download web pages, online live voice over Internet (e.g. SKYPE) connections are slow and prone to interference and eHealth applications which require the upload of data to a central collection point may not work because the broadband infrastructure cannot cope with the data transfer requirements of the application. Health care professionals and older adults complained about technological challenges:

“*… but unfortunately it* [the eHealth device that was trialled in the island community*] can’t connect to the phone lines, although it can take the data it can’t transmit it back*” (HP2, community nurse).

*Without technology* Interviewee 1’s spouse, who participated in the interview with his wife, talked about using websites devoted to the medical condition she suffered from and said “ *… a problem here is that the internet is so slow so you’ve got to have time to sit and let it - it can take two or three minutes for a page to load …”.*

Despite having limited personal experience of using eHealth, the health professionals we interviewed were positive about its use. They thought that eHealth helped to empower patients, allowing them to exert some control over their medical condition and to do as much as they could for themselves, by themselves. However, some words of caution were expressed. Health Professional 4, a community nurse, told us that, in her opinion, for eHealth to be beneficial, the user needs to be willing to learn, computer literature, have good backup from family and friends and, importantly, willing and able to take responsibility for their own care. These observations suggest that eHealth might not be the best care option for everyone. Introducing the use of new technologies across the board might not always be in the best interests of patients.

## Discussion

Key points from the analysis of responses to the Pain Association questionnaire are that respondents who were older and who lived alone (who were single, widowed or separated/ divorced) were the most likely to be in receipt of formal help from health and social care professionals and to use technology as part of their health care. Respondents who are younger and who live with others are most likely to receive informal help, and are less likely to receive formal help or to use technology as part of their health care. This type of care appears to negate both the need for that individual to require in-person visits from health and social providers and the use of technological help which, combined, reduce potential care costs borne by the state. Demographic ageing will increase the absolute numbers of older people and those living alone (e.g. after widowhood). This will have implications for care as it is unlikely that current levels of informal care per individual provided by family members (and friends and neighbours) can be continued [25,26]. However, it should also be noted that those individuals in receipt of informal help could potentially be unaware of technological aids. Health professionals could play a role in alerting their patients to what devices and applications are available and which might be useful in specific cases.

It is likely that the formal and technological help that is received by individuals who are older and living alone helps to maintain their independence and enables them to remain live at home (although only significant at 90% it is worth noting that respondents to the questionnaire who lived in rural areas were less likely than their urban counterparts to be using technology as part of their health care). The small number of rural respondents makes it inappropriate to infer too much from this finding, but given what is known from other research and in light of the observations made by health professionals interviewed in this remote island case study area about the challenges of using eHealth technologies, this finding highlights the need to ascertain whether, in rural areas, lower use of technology exists because the technology cannot be used due to infrastructure constraints rather than because patients are not willing to use it.

Key findings from the interviews with patients include the fact that those who had completed the *Pathways through Pain* programme liked being able to work through it at their own pace and valued the multi-professional delivery mode of the programme. *Pathways’ through Pain*’s inclusion of steps led by specialists from different health care professions is particularly important for people living in rural areas where local specialist services, private or NHS, do not exist. This attribute of *Pathway through Pain* demonstrates the potential eHealth could have to improve the accessibility of specialist services to rural areas.

Findings from both stages suggest that many older people are digitally literate and open to the use of technology as part of their chronic pain care package. This bodes well for Government plans to make eHealth initiatives more ubiquitous.

### eHealth: inclusion and exclusion

Findings from the two research stages both highlighted the potential of eHealth, but also suggest that individual preferences may influence whether or not health is a successful option for patients. It may be empowering for some yet exclusionary for others. Previous research has shown that a lack of accessibility can cause social exclusion in rural areas, whereas in urban areas a lack of accessibility is often a consequence of social exclusion [27]. Thus in rural areas mechanisms which could enhance accessibility could also promote inclusion. eHealth could empower individuals if their use promoted feelings of independence and enablement in the user. Further deployment of eHealth technologies could increase the accessibility of different services and improve the care options available to patients, again promoting social inclusion. Medium to long term benefits of engaging with eHealth were questioned by some of our participants. Whilst participating in the *Pathway through Pain programme* empowered interviewees for the duration of their enrolment, a couple were frustrated about being unable to return to resource or instructional materials, for example to be reminded how to perform a relaxation technique or a pain relieving set of exercises, once they had completed the programme (although, as noted above, it should be possible to return to these materials). There is therefore the potential that people could be left worse off, feeling “dis-enabled”, if they do not retain access to something they think has been of benefit to them.

### Not everyone is a willing or a suitable user of eHealth technologies

A Canadian study [28] noted that rural older men had smaller social networks than women and men used health and other services less than women. These findings help to explain our Stage 1 finding that, controlling for age, women were less happy to accept the use of technology as a formal element of their care in the future than men. Women have larger social networks and are, arguably, more sociable and value opportunities for social interactions more than men. Women do not want to countenance changes to their care that could result in fewer opportunities for social interaction. Male respondents, on the other hand, had smaller social networks and did not appear to be concerned about the loss of social interaction opportunities the use of eHealth might bring. Gender differences were difficult to explore in Stage 2 because, by chance, most of the participants were female. However, all our interviewees were users of technology. The gender differences reported in the Stage 1 questionnaire could reflect a female preference for in-person health care rather than an outright rejection of eHealth and other technologies. Men may be more amenable to the use of eHealth in the future because they are not as concerned about it replacing the social interaction opportunities in-person formal care provides.

The number of people living in a household is associated with the level of acceptance of care being provided by technology. Respondents who lived alone had higher levels of acceptance. This suggests that acceptance may relate to existing levels of personal and social contact. These individuals do not appear to be concerned that technology is replacing other types of care delivery. We suggest that this is because technological help is not *perceived* to be replacing their care, which highlights the importance of the interactions that technology cannot provide and that acceptance of technology may increase if it is viewed in this way.

Our findings suggest that eHealth users need to be motivated and willing to learn, suggesting that not all personality types are likely to benefit equally from using eHealth technologies. Chronic pain is a condition that, as illustrated above, can create ergonomic challenges to using the devices eHealth technology users and can make concentrating for extended periods of time very difficult. Individuals who do not ‘match’ a user profile may not be offered eHealth and thus be denied access to the potential benefits the technology could bring. A characteristic of rural healthcare that could have implications for the deployment of eHealth technologies is the close patient-health professional relationships commonly found in small rural communities [29]. On the one hand this could mean that health professionals who know their patients are well placed to determine a patient’s suitability for using eHealth but they may err on the side of caution if they think that the person (as opposed to the patient) might suffer from a change in their care regime (e.g. no longer having regular home visits). Alternatively, if there is a poor personal relationship between a patient and a health professional there is a risk that eHealth could be deployed as a means of ‘getting rid’ of a problem patient.

### The digital divide and challenges in universal deployment of eHealth technologies

The UK is not unusual in a global context in having some areas (mostly large urban centres) with excellent digital connectivity and other areas (many rural communities which cover large geographical areas) with very poor digital infrastructure – the urban–rural *digital divide*. The UK government has committed to ensuring all households have access to a minimum standard of 2Mbit/s broadband service [30] it has been argued that this level of infrastructure will provide connections that are too slow for many online activities [31], including many existing, let alone under development, eHealth technologies. Despite plans for the roll out of superfast broadband across the UK (see, for example, [30]) including remote rural areas, as the pace of technological advances is so fast there remains a risk that rural patients will be excluded from the benefits eHealth technologies offer simply because of infrastructure variations nationwide. Service planners - normally based in large urban centres with excellent digital infrastructure - need to remember that over large geographical areas of the UK the digital infrastructure is not capable of supporting all types of eHealth. Consideration should also be given to alternative care options and assumptions not made that eHealth can be rolled out nationwide universally. Rural patients, who potentially have the most to gain from the improved access to health care that eHealth offers may be further excluded if the use of eHealth to manage conditions such as chronic pain becomes more widespread.

### eHealth: supplementing or replacing care?

Findings from both stages suggest that technology is already being used to supplement existing care. The presence of eHealth in the home of a single older person may provide reassurance to both the individual and their friends and family and may be a cost effective means of allowing the individual to remain living independently at home. However, many questions about future use of home-based technology in care remain unanswered. For example, if eHealth is used to supplement existing care, is eHealth used because it is perceived to be ‘added-value’ by patients? If so, this suggests that it has the potential to increase as well as decrease the cost of providing services to patients in different areas. Also, it is unknown whether the presence of eHealth in someone’s home may decrease in-person visits by health professionals. If visits are reduced could patients’ wellbeing suffer from a reduction in social interaction opportunities between professionals and patients? Stage two found that the usefulness of eHealth and whether it should be used as a standalone element of care or as part of a care package depends upon the severity of chronic pain experienced by individuals. As a patient’s condition deteriorates, eHealth can only be used to supplement other health care. We do not yet have standardised measures that could be used to determine what the most appropriate combination of eHealth and other types of care would be.

### Limitations of the study

We acknowledge some limitations of our study. Many people do not know about or chose not to join groups such as Pain Association Scotland and research which relies on third party membership lists to access a specific population of interest may not capture the attitudes and opinions of all individuals who fall within that group. However, we believe the limitations are outweighed by the benefits of being able to target a specific group - in this research - chronic pain sufferers. Only a small proportion of Pain Association Scotland members lived in a rural area thus the absolute number of rural respondents is small but no obvious alternative method of targeting the chronic pain population in Scotland, living in urban or rural areas, existed. The proportion of rural respondents (16.2%) broadly mirrored the urban/rural distribution of population in Scotland) and our analysis identified some statistically significant differences between urban and rural respondents. If the rural sub-sample had been larger other relationships may have emerged.

The interviewees in this study were all older rural adults who were receiving different forms of health care as a result of chronic pain. The island case study participants were all severely afflicted by chronic pain. Our findings, therefore, do not necessarily reflect the views of the many older people who do not receive the same levels of health or social care support. This would be difficult research group to identify but, nevertheless, it is worth highlighting that the views of unmanaged chronic pain sufferers are not reflected in this study.

## Conclusions

This study aimed to explore attitudes towards, the use of, and acceptance of technology in rural health and social care, focusing on people experiencing chronic pain who live in remote rural areas of Scotland. We found an overall openness to technology being used as part of a care package, but opinions about the extent to which technology should complement or replace existing modes of care differed. Men were more accepting than women. Those who were already in receipt of formal care were more accepting than those who did not receive formal care. Those who lived alone were more accepting that those who lived with at least one other person. Finally, those who did not have any relatives living nearby were more accepting than those who had relatives who lived closer to them. Acceptance of technology therefore relates to existing levels of personal and social contact, and appears to be greater where technological help is not perceived to be replacing in-person care. We predict that acceptance of eHealth is likely to increase as future generations of older people have more experience of using technology in everyday life. Currently those who do use technology are broadly open to using eHealth if it is to supplement the care they already receive. Our findings suggest that these positive opinions about eHealth could change if the technology is perceived to be replacing in-person visits from health and/or social care professionals. From our observation of the activities that occur during a home-visit [32], it is very unlikely that eHealth would replace the need for in-person visits for those most seriously afflicted by chronic pain.

eHealth has been heralded as a cost efficient solution to the service delivery challenges of providing care in rural communities. It has the potential to assist with both the management of some health conditions (illustrated in this paper with reference to those living with chronic pain), and to assist with tackling issues of service provision and accessibility to services for an ageing population/ increasing numbers of older people in more remote and rural communities. This paper suggests that before any widespread deployment of eHealth is instigated, it is imperative to understand problems older people might have in using and accessing eHealth. Rather, eHealth should be considered as being part of a suite of responses to provide more care options to patients. It has the potential to make services more easily available - particularly in rural areas - where it is often difficult for people to accessing mainstream services. Based on our research, we predict that in the future eHealth solutions will become mainstreamed in rural areas if as is predicted, the ageing population becomes more digitally literate and if broadband services are improved However, it should not be considered to be the answer for everyone.

Most people are afraid of change. We recommend that for eHealth roll out to be a success it is important for patients to feel that they have been individually considered, and deployment should begin by supplementing, not replacing, more traditional in-person visits. This would give the patient time to become familiar with eHealth and comfortable with having fewer home visits. It is also imperative that greater understanding is gained about who eHealth can support most effectively and at what stage of an illness it will have the most benefit. We stress that eHealth technologies are not the best care solution for everyone, but selective, carefully decided deployment would be of benefit to patients, their families and their carers.

### Endnotes

^a^A very broad concept which encompasses telehealth and telecare technologies “*Telecare involves monitoring aspects of an individual’s activity, or related activities in the home…Telehealth technologies, on the other hand, require active involvement from the user to take readings (e.g. blood pressure, respiratory measures, blood glucose, symptom measurement) and submit them to a remote clinician for expert review”. The submission of readings is often done online, thus the user needs to have an Internet connection* [1].

^b^Ethical approval for the survey research conducted via Pain Association Scotland, stage 1, was awarded by the University of Aberdeen. Ethical approval for the work undertaken in stage 2 was approved by a National Health Service Research Ethics Committee and locally at the level of individual NHS R&D Departments.

^c^In Scotland health services are delivered by fourteen National Health Service Boards which cover distinct geographical areas.

## Additional files

Additional file 1: Table S1. Significant associations for different types of help respondents were receiving. Additional file 2: Table S2. Significant findings for current use of, and attitudes towards future use of, technology in the home.
