# Supplementary material for: Attitudes towards the use and acceptance of eHealth technologies: a case study of older adults living with chronic pain and implications for rural healthcare
Source: BMC Health Serv Res. 2015 Apr 16;15:162. doi: 10.1186/s12913-015-0825-0 (PMC4415301; doi:10.1186/s12913-015-0825-0)
Supplement: Additional file 1: Table S1. — Significant associations for different types of help respondents were receiving. [file 12913_2015_825_MOESM1_ESM.docx]

|  | **Receives formal help (from health and social care professionals)** | **Receives informal help from friends and family** | **Uses a health monitoring device** |
| --- | --- | --- | --- |
| **Age** | *Those aged 60+ were more likely to receive formal help than younger adults*  *x*^2^ = 5.971, df = 2, p = 0.05, |  | *Those aged 60+ were the more likely to use health monitoring devices than younger adults*  *x*^2^ = 6.925, df = 2, p = 0.03 |
| **Place of residence: urban or rural** |  |  |  |
| **Number of people in the household** |  | *Respondents who live alone were less likely to receive informal help than those who live with others*  *x*^2^ = 5.910, df = 2, p = 0.05 |  |
| **Respondent is retired** |  |  | *The retired were more likely than those in other economic activity categories to use health monitoring devices*  *x*^2^ = 3.878, df = 1, p = 0.05 |
| **Respondent is permanently sick** |  |  |  |
| **Life satisfaction** |  |  |  |
| **Living with spouse** |  | *Respondents who live with their spouse were more likely to receive informal help than those who live alone.*  *x*^2^ = 5.971, df = 2, p = 0.05 |  |
| **Respondent now living alone^[[1]](#footnote-1)^** | *Respondents who now live alone were more likely to receive formal help than those who have always lived alone or who live with their spouse.*  *x*^2^ = 12.004, df =1, p = 0.00 |  | *Respondents who now live alone were more likely to use health monitoring devices than those who have always lived alone or who live with their spouse.*  *x*^2^ = 4.824, df = 1, p = 0.03 |
| **Respondent has always lived alone ^[[2]](#footnote-2)^** | *Respondents who have always lived alone were less likely to receive formal help than those who now live alone or those who live with their spouse*  *x*^2^ = 4.924, df = 1, p = 0.03 |  |  |

|  | Significant results (p≤0.05) |
| --- | --- |
|  | Almost significant results (p>0.05) |

1. Respondents now living alone are those who used to live with a partner but are now widowed or separated / divorced. [↑](#footnote-ref-1)
2. Respondents who have has always lived alone are single but not through divorce/ separation or widowhood. [↑](#footnote-ref-2)
