# Supplementary material for: Attitudes towards the use and acceptance of eHealth technologies: a case study of older adults living with chronic pain and implications for rural healthcare
Source: BMC Health Serv Res. 2015 Apr 16;15:162. doi: 10.1186/s12913-015-0825-0 (PMC4415301; doi:10.1186/s12913-015-0825-0)
Supplement: Additional file 2: Table S2. — Significant findings for current use of, and attitudes towards future use of, technology in the home. [file 12913_2015_825_MOESM2_ESM.docx]

|  | **Uses the Internet** | **Is happy to consider using technology for healthcare in the future** | **Would accept technology replacing in-person visits from health professionals in the future** | **Would accept technology replacing home help visits in the future** |
| --- | --- | --- | --- | --- |
| **Age** | *Respondents aged under 60 were more likely than those aged over 60 to use the Internet*  *x*^2^ = 8.677 , df = 2, p = 0.01 |  |  |  |
| **Sex** |  | *Males were more happy to consider using technology for healthcare in the future than females*  *x*^2^ = 7.321, df = 2, p = 0.03 |  |  |
| **Number of people in the household** |  |  |  |  |
| **Number of relatives living in the local area** |  | *Respondents with no relatives in the local area were happier to consider using technology for healthcare in the future than respondents without relatives living nearby*  *x*^2^ = 4.924, df = 4, p = 0.05 |  |  |
| **Respondent is retired** | *Retired respondents were less likely to use the Internet than those in other economic activity categories*  *x*^2^ = 6.760, df = 1, p = 0.02 |  |  |  |
| **Respondent now living alone^[[1]](#footnote-1)^** | *Respondents who now live alone are less likely to use the Internet than those who have always lived alone or who with their spouse*  *x*^2^ = 4.740, df = 1, p = 0.03 | *Respondents who now live alone are less likely to consider using technology for healthcare in the future than those who have always lived alone or with their spouse*  *x*^2^ = 9.266, df = 2, p = 0.01 |  |  |
| **Living with spouse** |  |  |  |  |
| **Respondent has always lived alone** | *Respondents who have always lived alone are more likely to use the Internet than those who have always lived alone or who now live with their spouse x*^2^ = 4.6-5, df =1, p = 0.03 | *Respondents who have always lived alone are more happy to consider using technology for healthcare in the future than those who have always lived alone or now live with their spouse.*  *x*^2^ = 6.806, df = 2, p = 0.03 |  |  |

|  | Significant results (p≤0.05) |
| --- | --- |
|  | Almost significant results (p>0.05) |

1. Respondents now living alone are those who used to live with a partner but are now widowed or separated / divorced. [↑](#footnote-ref-1)
